# Heat Stress Mitigation Strategies in Feedyards: Use, Perceptions, and Experiences of Industry Stakeholders

**DOI:** 10.3390/ani13193029

**Published:** 2023-09-26

**Authors:** Lauren Dean, Anthony J. Tarpoff, Kirsten Nickles, Sara Place, Lily Edwards-Callaway

**Affiliations:** 1 Department of Animal Sciences, Colorado State University, Fort Collins, CO 80523, USA; lauren.dean@colostate.edu (L.D.); sara.place@colostate.edu (S.P.); 2 Department of Animal Science and Industry, Kansas State University, Manhattan, KS 66506, USA; tarpoff@ksu.edu; 3 Certified Angus Beef LLC, Wooster, OH 44691, USA; knickles@certifiedangusbeef.com

**Keywords:** cattle, heat stress, feeding, feedlot, producer, perception, shade, sprinklers

## Abstract

**Simple Summary:**

In the United States, cattle in feedyards can experience heat stress due to extreme climatic events negatively affecting welfare and productivity. Various heat mitigation strategies have been studied in feedyard settings, but information on the current adoption of those strategies and stakeholder perceptions of strategies and their benefits is limited. This study utilized an online survey to ask feedyard operators, veterinarians, and nutritionists about their perception and use of heat mitigation strategies. Generally, respondents agreed that heat stress impacts cattle performance, health, and welfare. The majority of respondents utilized or recommended some type of heat mitigation such as changing feed delivery time and feed composition, utilizing sprinklers and/or changing processing and handling times. Respondents used a variety of indicators and monitoring tools to aid in identifying when to implement protocols and strategies. Respondents perceived pen infrastructure (i.e., shade, fans) and water/feed management as important considerations for better mitigating the negative effects of heat stress on cattle in the face of another extreme heat event. This survey highlighted the need for future research to evaluate the cost:benefit of different mitigation strategies, the effectiveness of mitigation strategies in different geographical areas, and development of more in-depth heat stress forecasting and monitoring tools.

**Abstract:**

The purpose of this study was to: (1) understand heat mitigation strategies currently used and recommended by feedyard operators, veterinarians, and nutritionists, (2) understand their perceptions of heat mitigation strategies related to cattle health, performance, welfare, and carcass quality, (3) quantify the frequency of extreme heat events, and (4) understand industry needs associated with heat stress mitigation strategies. An online survey was shared via 11 industry association listservs. Descriptive statistics were performed on 56 responses (n = 22 operators, 26 veterinarians and eight nutritionists). Thematic analysis was performed on free-response questions. Sixteen (72.7%) operators, 23 (88.5%) veterinarians and eight (100%) nutritionists utilized at least one heat mitigation strategy. “Changing processing and shipping hours” (n = 42, 75%) had the most “strongly agree” responses when asked about strategy effectiveness. The majority of respondents agreed that heat stress negatively impacts cattle health, performance, and welfare (Mean ± SD; ≥7.8 ± 2.6 for all roles). Forty-two (75%) respondents experienced cattle death loss from extreme heat events. Thematic analysis indicated that respondents perceived pen infrastructure and water/feed management as important considerations for better mitigating heat stress impacts. When asked what resources would be helpful, respondents indicated research and data regarding the effectiveness of various strategies.

## 1. Introduction

The detrimental effects of heat stress on cattle welfare and productivity are well established (reviewed by [1,2,3]). In hot conditions, when heat accumulation surpasses heat dissipation, cattle undergo physiological and behavioral changes to regulate body temperature that can negatively impact growth [4,5], milk production [6], reproduction [7], and immune function [8], among other parameters. With climate changes, heat events are becoming more frequent, more intense, and longer in duration [9,10], thus creating new challenges for cattle production globally [11]. In the United States, this climate variability is coupled with an ever-growing demand for cattle efficiency and growth [12], emphasizing the importance of finding effective ways to mitigate heat stress for cattle in production facilities. Finding solutions to managing the detrimental effects of heat stress on cattle welfare and productivity is critical to the future sustainability of the cattle industry in the United States. Cattle producer education programs, primarily the National Cattlemen’s Beef Association (NCBA) Beef Quality Assurance (BQA) program, provide some best management practices for proactively addressing heat stress risks in cattle primarily related to adjusting cattle handling and transport in extreme weather [13]. In some third-party verified animal care audit programs, there are requirements related to heat stress mitigation, but they tend to focus on ensuring cattle have protection from extreme weather (both cold and hot), primarily through the use of shade [14] and/or the use of water cooling systems (i.e., sprinklers [15]) and not other types of heat mitigation. Overall, there are limited specific guidelines for managing heat stress in cattle likely because the need for mitigation varies across production systems (i.e., region, time of year, management approach, and cattle type) in addition to the fact that there are many different strategies that could be implemented, making it challenging to be prescriptive.

Several strategies exist that help reduce the impacts of heat stress on cattle in feedyards and are often categorized into adjusting feed, water, environment modifications, or handling changes [16]. The effectiveness of heat mitigation strategies has been studied in feedyard settings to varying degrees (e.g., shade, reviewed by [17]), but information on the current adoption of those strategies and stakeholder perceptions of their effectiveness are limited. Much of the current literature has been focused on certain regions within the United States. Both Simroth et al. [18] and Samuelson et al. [19] reported on the use of shade in feedyards in specific regions of the United States. Both these studies indicated that less than 20% of respondents utilized shade in home pens on feedyards, a relatively low percentage considering the noted benefits of shade use [17]; the use of shade in hospital pens was greater (47%), as reported in Simroth et al. [18]. Rusche et al. [20] conducted a recent survey in which feedyard operators from three states (MN, NE and SD) were asked about their use and perceptions of a variety of heat stress mitigation strategies. Respondents indicated variable adoption of a wide variety of techniques (e.g., adjusting feeding delivery and altered shipping schedule) and perceptions of successful and less successful strategies. Some types of heat mitigation are studied more frequently than others and understanding stakeholder perceptions about the effectiveness of currently used practices, limitations on the adoption of new practices, and needs regarding preparing for heat events and heat stress monitoring tools would be valuable for addressing current industry needs.

Most of the previous research on heat stress mitigation strategy adoption has included either feedyard operators and/or nutritionists [18,19,20]. The vast majority of feedyards (95.2% of feedyards greater than 1000 cattle) included in the 2011 NAHMS Feedlot study reported using the services of a nutritionist [21]. Several heat stress mitigation strategies relate to feeding management (i.e., number of feedings and times of feedings) and feed content (e.g., additives or changing diet content), thus highlighting the value of including a nutritionist in discussions about cattle management before, during, and after extreme heat events. The majority of feedyards also consult with veterinarians; the 2011 NAHMS Feedlot study reported that nearly all feedyards greater than 1000 cattle (96.6%) used the services of a veterinarian in some capacity to assist with ensuring cattle health [21]. Despite the widespread involvement of veterinarians in cattle health decisions, the current research on heat stress mitigation strategies has not consistently included veterinarians in the evaluation of management practices and their impact on cattle health and welfare. It is critical to include veterinarians in future evaluations of and discussions about managing heat stress in feedyard cattle.

The cattle industry has suffered significant cattle death loss in recent years due to the increasing number of extreme heat events [22,23]; in the summer of 2022, the Midwest United States experienced an extreme heat event that was responsible for the deaths of thousands of feedyard cattle [24]. Outside of popular press articles, little is known about the frequency with which feedyard operators experience death loss due to heat stress. The NAHMS 2015 Death Loss Report indicated that 9.3% of cattle death loss was attributed to weather-related issues by participants; the specific weather issue was not reported and thus was not specific only to extreme heat [25]. Death loss has obvious negative impacts on cattle welfare but additionally affects the profitability of feedyard operations. The most recent analysis of economic loss due to heat stress in beef cattle indicated a $369 million annual loss attributed to reductions in cattle growth and reproduction and increases in mortality [26]. This is likely a conservative estimate, as many factors have changed since this analysis (i.e., changes in weather, markets, cattle characteristics) that may impact the economic loss associated with heat stress in cattle.

The purpose of this study was to: (1) understand heat mitigation strategies currently used by feedyard operators, veterinarians, and nutritionists, (2) understand their perceptions of heat mitigation strategies as it relates to cattle health, performance, welfare, and carcass quality impacts, (3) quantify the frequency of extreme heat events, and (4) understand industry needs and challenges associated with heat stress mitigation strategies.

## 2. Materials and Methods

Research protocols were reviewed and deemed exempt by Colorado State University (CSU) Institutional Review Board (#3797) prior to project initiation.

### 2.1. Study Population and Recruitment

The target population for this study included feedyard operators, nutritionists, and veterinarians who worked at or with feedyards. Eleven associations were asked to distribute the survey to their listservs via email with a brief overview of the content and goal of the project. Participating associations included (n = total members for each organization): Beef Alliance (n = 9 ), Texas Cattle Feeders Association (n = 175), Kansas Livestock Association (n = 192), Colorado Livestock Association (n = 72), American Association of Bovine Practitioners (n = 1617), Academy of Veterinary Consultants (n = 800), National Cattlemen’s Beef Association Beef Quality Assurance State Coordinators (n = 80), California Cattlemen’s Association (n = 35), Nebraska Cattlemen (n = 2600), North Dakota Stockmen’s Association (n = 2000), and Oklahoma Cattlemen’s Association (n = 3500). The survey link may have been shared with additional individuals by listserv members. Responses were collected between 1 November 2022 and 2 January 2023. Reminder emails may have been distributed during this time period and varied by group.

### 2.2. Survey Development and Content

Researchers from CSU and Kansas State University (KSU) created questions for this survey. Researchers shared the survey draft with several industry representatives to receive feedback on relevancy and clarity of questions. An online platform (Qualtrics, Provo, UT, USA) was used to deliver the survey. Prior to distribution, all collaborators reviewed the questions for accuracy and clarity. Operators, veterinarians, and nutritionists received different question tracks; questions for veterinarians and nutritionists were further branched into questions for veterinarians and nutritionists who worked with only one feedyard and those who worked with multiple feedyards. Operators were asked 55 questions, veterinarians were asked 44 or 45 questions depending on the number of feedyards they worked with, and nutritionists were asked 43 or 44 questions depending on the number of feedyards they worked with; some questions had additional follow-up questions if the respondent wanted to share more. The survey was divided into 10 sections: Facility and Cattle Description, Heat Stress Mitigation Strategies-General, Water, Management, Pen Management, Pen Construction, Nutrition, Perceptions, Extreme Heat Events, and Demographics. The survey included multiple choice, multiple response, Likert scale, and free-response questions. The Likert scale questions were asked using a 0 to 10 scale; one set of questions asked about effectiveness, 0 representing “Not Effective” and 10 representing “Extremely Effective”, and two sets of questions asked about agreement, 0 representing “Strongly Disagree” and 10 representing “Strongly Agree”. No identifying information was collected, and all survey responses were anonymous. All questions, apart from consent, were optional and respondents were not offered any type of incentive for survey completion. The survey can be found in Supplementary Material (Document S1: Survey Questions).

### 2.3. Statistical Analysis

A total of 125 surveys were received. Seven were removed because they did not represent one of the target roles (i.e., operator, veterinarian, or nutritionist). Sixty-two were removed because they were less than 80% complete; many of these were less than 50% complete. A total of 56 surveys were included in the final analysis. The response rate for included surveys was calculated to be approximately 0.51% based on the listserv memberships provided by associations; however, accuracy of the response rate is difficult to determine because survey recipients may not have met the requirements to participate (i.e., not a feedyard operator, etc.). Summary statistics were completed in Microsoft Excel for a selection of questions. Operator, veterinarian, and nutritionist results were summarized as separate groups. Unless stated otherwise, operators were asked to answer questions based on current practices at the feedyard with which they were affiliated, whereas veterinarians and nutritionists were asked if they recommended the specific strategies. Cattle demographic responses are only reported for the operator respondents; due to the nature of how veterinarians and nutritionists work with multiple feedyards, these questions were asked slightly differently and were therefore challenging to summarize together. The presence of shade in pens was categorized as “yes” or “no” for reporting purposes. Likert scale responses were collapsed into categories for presentation for questions asking about effectivity of strategies and impact of heat stress on different outcomes. The following categories were used: rating 0 to 3, Low; rating 4 to 7, Middle; and rating 8 to 10, High.

Thematic analysis was performed on the following three free-response questions: “Please share what you would do differently in the face of another extreme heat event”; “What resources would be helpful to you in regard to managing heat stress? Please explain”; and “How does dealing with an extreme heat event impact you and/or your employees? What are the most difficult aspects of dealing with an extreme heat event? Please explain”. Three researchers, two of whom are authors of the current study (L.D. and L.E.-C.), familiarized themselves with the qualitative data from the respondent responses, identified patterns (i.e., themes) that were present within the responses, and developed themes. Each coder reviewed responses and coded each response using the set of themes agreed upon by the three coders.

Partial agreement occurred, typically with multi-themed responses due to one coder missing or having additional code selection, and was discussed until full agreement was reached between the three coders. One coder considers herself an animal welfare scientist currently conducting industry-relevant research related to beef cattle welfare and has past experience working in the livestock industry. The second coder is working on his Master’s degree in Animal Science, focusing on genetics, and has experience working in the livestock industry. The third coder is a Master’s student, also in Animal Sciences, focusing on livestock welfare, and has experience working in the livestock industry.

## 3. Results

### 3.1. Respondent Demographics, Cattle Characteristics, and Facility Description

Respondent demographics are shown in Table 1. Slightly less than half of the respondents were veterinarians (n = 26, 46.4%) followed by feedyard operators (n = 22, 39.3%) and nutritionists (n = 8, 14.3%). A majority of respondents identified as men (n = 46, 82.1%) and white (n = 48, 85.7%). Ages of respondents ranged from 22 to 80 years old, with the greatest representation being from age 40 to 59 years of age (n = 24, 42.9%). More than half (n = 36, 64.3%) of the feedyards represented were based in the Midwest. There was one respondent from Canada whom the authors decided to keep in the data set, as the respondent was affiliated with one of the listservs. Veterinarians and nutritionists overseeing more than one feedyard were able to select all regions in which they practiced or consulted, so the regional total is greater than the total respondents.

Table 2 includes cattle characteristics from operators only; veterinarians and nutritionists often worked with multiple feedyards, so these questions were not asked of all survey respondents. British breeds had the greatest representation (n = 18, 81.8%), as well as black-hided cattle (n = 22, 100%), although many other breeds and hide colors were present at the feedyards represented. Almost all operators had both steers and heifers at their facilities (n = 21, 95.5%). The average one-time capacity reported by operators ranged from 50 to 125,000 cattle, with a majority of operators reporting greater than 1000 cattle on their feedyard (n = 14, 63.6%). The total number of cattle represented by operator respondents was 559,409.

Respondents were asked various questions about their facilities. The majority of feedyards (n = 42, 75.0%) reported only outside (i.e., uncovered) pens. There were several that had a combination of both indoor and outdoor pens (n = 10, 17.9%) or indoor only pens (n = 4, 7.1%). Approximately half (n = 31, 55.4%) of the respondents reported having shade in at least some home pens, three-quarters (n = 43, 76.8%) in hospital pens, and half (n = 28, 50.0%) in holding pens.

### 3.2. Heat Stress Mitigation Strategies

When asked about implementation of heat stress mitigation strategies, 72.7% (n = 16) of operators, 88.5% (n = 23) of veterinarians, and 100% (n = 8) of nutritionists answered that they utilize or recommend heat mitigation strategies. However, when asked if they had a written protocol for describing heat stress mitigation strategies, only 22.7% (n = 5) of operators selected yes; a majority of veterinarians (n = 17, 65.4%) and nutritionists (n = 5, 62.5%) selected yes to having a recommended written protocol. Table 3 shows the use/recommendation of various heat stress mitigation strategies by respondent role.

Utilizing water as a type of mitigation strategy was the least utilized by operators: 36.4% (n = 8) added additional water availability, 22.7% (n = 5) utilized a sprinkler system, and 22.7% (n = 5) watered cattle down. However, a majority of veterinarians and nutritionists recommended adding additional water availability (n = 23, 88.5%; n = 7, 87.5%, respectively) and utilizing a sprinkler system (n = 16, 61.5%; n = 6, 75%, respectively).

Changing work hours during an extreme heat event was implemented by 72.7% (n = 16) of operators and recommended by all veterinarians and nutritionists. The change of processing/re-implanting/shipping times was implemented by 91% (n = 20) of operators and recommended by 96.2% (n = 25) of veterinarians and all nutritionists. The majority of the operators modified feeding strategies during (n = 12, 54.5%) and after (n = 16, 72.7%) extreme heat events; similarly, most veterinarians and nutritionists also recommended to modify feeding strategies during (n = 20, 76.9%; n = 8, 100%, respectively) and after (n = 17, 65.4%; n = 5, 62.5%, respectively) extreme heat events. For operators who responded yes to modifying feed strategies during an extreme heat event, most indicated that they changed feed times (n = 8, 66.7%%), but in response to modifications of feed after an extreme heat event they most often changed feed times (n = 8, 50%) as well as feed composition (n = 8, 50%). Both veterinarians and nutritionists commonly recommended changing feed delivery time during extreme heat events (n = 16, 80%; n = 5, 62.5%, respectively). For those veterinarians and nutritionists who recommended changing feeding strategies after an extreme heat event, veterinarians most often recommended changing feed times (n = 12, 70.6%) and feed composition (n = 12, 70.6%), while nutritionists most often recommended changing feed times (n = 4, 80%).

### 3.3. Heat Stress Indicators and Heat Event Monitoring Tools

All survey respondents were able to select more than one option in response to the questions about indicators and tools used to determine the need for implementing mitigation strategies; therefore, percentages are greater than 100% (Table 4). Among indicators used to determine implementation of heat stress mitigation strategies, operators most often used “daily forecast” (n = 19, 86.4%; Table 4), followed by “multiple day heat event anticipated” (n = 18, 81.8%) and “cattle behaviors” (n = 17, 77.3%). Veterinarians and nutritionists most often chose “multiple day heat event anticipated” (n = 23, 88.5%; n = 7, 87.5%, respectively), with “cattle behaviors” and “daily forecast” also being in the top three indicators selected across both stakeholder groups. The top selection for each respondent role in response to what heat event monitoring tool they use was: “local forecast” for operators (n = 19, 86.4%) and “temperature humidity index” for both veterinarians (n = 19, 73.1%) and nutritionists (n = 6, 75%).

### 3.4. Extreme Heat Events

Forty-two (75%) respondents had experienced cattle death loss from extreme heat events. Of the respondents who answered yes to experiencing cattle death loss from extreme heat events, the months reported with the greatest death loss (respondents could select more than one month) were July (n = 32, 76.2%), followed by August (n = 28, 66.7%). When asked the severity of loss (i.e., minor, moderate, severe), half of the respondents explained their perception of loss as minor; no definition was provided for the different levels of loss, so this response was based on respondent interpretation. When asked to explain what loss meant, most responses characterized loss by number of cattle that died or the number of events experienced per year.

### 3.5. Perceptions of Heat Stress Mitigation Strategies, Supportive Resources, and Heat Stress Impacts

Likert scales were used for a series of statements regarding perceptions of heat stress mitigation strategy effectiveness (Table 5). Overall, “changing processing and shipping hours” as a heat stress mitigation strategy had the greatest mean effectivity selected of all statements, across respondent role (Mean ± SD; operator, 8.0 ± 2.2; veterinarian, 8.6 ± 1.4; nutritionist, 8.8 ± 1.7); this strategy also had the fewest respondents in the low-effectivity category (only one respondent selected a 3 or below; Table 5). The effectiveness of “providing shade” at minimizing the effects of heat stress on cattle was also rated relatively high on the scale (Mean ± SD; operator, 7.1 ± 3.1; veterinarian, 8.7 ± 1.2; nutritionist, 8.6 ± 1.9). The strategy with the lowest mean effectivity selected was “using a sprinkler system” (Mean ± SD; operator, 5.2 ± 3.3; veterinarian, 6.1 ± 2.9; nutritionist, 5.5 ± 2.9). This strategy also had the largest number of respondents (n = 14) that did not provide an answer.

Likert scales were used for a series of statements regarding perceptions of supportive resources (Table 6). The mean agreements across respondent groups were similar and in the middle of the agreement scale for both the statement “the triggers I use to begin implementing heat stress mitigation work” and “the heat stress monitoring tools I use are helpful”. When asked if “involving a veterinarian in heat stress management decisions is helpful”, the veterinarian respondents had the greatest level of agreement (Mean ± SD, operators, 5.9 ± 3.7; veterinarians, 8.3 ± 1.6; nutritionists, 5.0 ± 1.5). The mean agreement was numerically greater across veterinarian and nutritionist respondents in response to the statement “involving a nutritionist in heat stress management decisions is helpful” (Mean ± SD; operator, 6.2 ± 3.6; veterinarian, 8.0 ± 2.1; nutritionist, 7.5 ± 2.3). There were several operators that selected a 3 or lower (low agreement) to both statements.

Likert scales were used for a series of statements about the impact of heat stress on cattle performance, health, welfare, and carcass quality (Table 7). The mean ratings for “heat stress negatively impacting cattle” performance, health, and welfare were all relatively high (greater than 7.8 for all respondent roles). The statement about “heat stress negatively impacting carcass quality” had the lowest mean agreement across roles (Mean ± SD; operator, 5.8 ± 2.9; veterinarian and nutritionist, 7.3 ± 2.3), all means falling within the middle agreement category. The carcass quality statement also had the most responses within the low agreement category (n = 6), in addition to the largest number of individuals not providing a response (n = 5).

### 3.6. Thematic Analysis

Thematic analysis was performed on three free-response questions. Eleven themes were identified and used to code question responses to all questions: Water and Feed Management, In-Pen Infrastructure, Cattle Characteristics, Cattle Movement Strategies, Preparation, Research and Data, Cost, Morals, Physical Stress, Monitoring Resources, and Other (Table 8). Definitions, examples, and the questions in which the theme was present are shown in Table 8. The Other theme was present only a few times and included comments that did not fit within the other categories.

#### 3.6.1. Question 1: Please Share What You Would Do Differently in the Face of Another Extreme Heat Event

The two most common themes found in responses to this question were Water and Feed Management and In-Pen Infrastructure, and these themes were often coded with the theme of Preparation. The majority of the comments representing Water and Feed Management referred to altering feeding strategies, for example, “reduce morning feed delivery”, and “change ration up to 2 days before event”, or providing more water availability, for example “…water water water”, and “…add increase water availability via additional tanks OR loose up the number of cattle per available waterer”. Many of the responses coded as In-Pen Infrastructure either referred to providing bedding (e.g., “…utilize bedding” and “be more proactive in providing bedding…”) or providing shade (e.g., “make sure shades are up…” and “Shade in high risk cattle…”). Cattle Characteristics and Cattle Movement Strategies were also mentioned but less frequently, and the responses within these categories all focused on things to do differently or sooner.

#### 3.6.2. Question 2: What Resources Would Be Helpful to You in Regard to Managing Heat Stress? Please Explain

The most common theme in the responses to this question was Research and Data. Respondent responses were centered around wanting more research or data generally (e.g., “Good solid data” and “Data from well-managed trials”) with some examples of specific needed areas of research. These types of responses were often also coded with another theme, such as In-Pen Infrastructure. For example, some respondents shared: “Large pen research showing impacts of shade, irrigation, or other types of mitigation…”, “Research on the use of shades on the high plains”, and “What feed additives help with heat stress? I have mineral with garlic but that’s the only one I’m aware of”. The theme of Cost also was found more frequently than some of the others and included comments such as: “More money”, “Cost analysis to show economic value of shade placement”, and “Shade trials and funding to help with shade construction”.

#### 3.6.3. Question 3: How Does Dealing with an Extreme Heat Event Impact You and/or Your Employees? What Are the Most Difficult Aspects of Dealing with an Extreme Heat Event? Please Explain

The most common themes in the responses to this question were Physical Stress, Morale, and Cost. Comments coded as Physical Stress were focused on the impacts that the heat has on employees (e.g., “Them having heat stress just like the animals” and “hard on staff working in heat”). In addition to impacts on physical stress, emotional aspects were also mentioned. The responses coded as Cost most often referred to either financial loss associated with heat stress and the extra time needed to manage animals.

## 4. Discussion

There is a considerable amount of research demonstrating the negative impacts of heat stress on the welfare and productivity of cattle in feedyards (reviewed by [1,2,3]). There is limited information detailing the adoption of some key heat mitigation strategies utilized by feedyard operators. The few studies that do report statistics on certain heat abatement provisions are limited in number and have been focused on specific regions within the United States [18,19,20]. These studies have also demonstrated variable adoption rates of some common heat stress mitigation strategies. There is also little data available indicating what the perceptions are of heat stress management strategies, and understanding this could help explain why some strategies are or are not being implemented, in addition to identifying knowledge and research gaps. Additionally, many of the current research studies have focused on feedyard operators (or managers) and/or nutritionists [18,19,20]; veterinarians have not been included as key stakeholders in many of these studies. The intent of the current study was to survey key feedyard industry stakeholder groups (i.e., operators, nutritionists, and veterinarians) to understand what current heat mitigation strategies are being implemented, what the perceptions are of those strategies, and ultimately understand current industry needs and challenges associated with this important topic that has impacts on the sustainability of cattle production systems.

This survey was distributed nationwide to a variety of organization and association membership email lists and listservs. Unfortunately, despite the broad recruitment strategy, the response rate for the survey was low, which limits the overall conclusions that can be made from this study. Response rates to online surveys are highly variable and can be influenced by many factors, including accessibility to internet, survey length, use of reminders, and salience of the issue [27]. The current survey was lengthy, as the hope was to gain some detailed information about how operations are using a variety of heat mitigation strategies. Additionally, the reminder schedule varied by organization so some groups did not receive follow-ups which could have impacted overall response rate, although authors feel this impact would be marginal. The negative impact of heat stress on cattle welfare and productivity is certainly a salient topic within the cattle industry, but perhaps the timing of survey administration (e.g., during a time when there was no risk of extreme heat events) influenced the importance respondents associated with the issue. Future endeavors to understand feedyard stakeholder perspectives on heat stress mitigation could be performed in conjunction with industry and association meetings in a focus-group format which would provide overarching opinions around this topic and could help streamline future survey efforts. Despite the small sample size, the findings from this study can still be used to identify some key areas for further exploration.

Survey respondents represented three critical stakeholder groups: feedyard operators, nutritionists, and veterinarians. The majority of respondents were either feedyard operators or veterinarians that consulted with feedyards; nutritionists only made up a small proportion of the study population. As nutritionists play a key role in managing cattle health and some of the included heat mitigation strategies relate directly to feeding, it is important to try different ways to engage this interest group. Respondents represented feedyards mostly in the Midwest region of the United States, which includes top cattle-producing states [28]. Although the survey population was relatively low, the number of cattle represented by operator respondents was approximately 4.8% (n = 559,409 head) of the feedyard cattle population in the United States; the USDA June Cattle on Feed report estimated 11.6 million cattle and calves on feed in US feedyards with a capacity of 1000 or more head [28]. Again, future work should adopt recruitment strategies (e.g., attending regional meetings, local advertisements) to be able to include operations of varying size (i.e., number of cattle) and location within the United States in addition to targeting individuals who provide supportive services, such as nutritionists and veterinarians.

The majority of survey respondents indicated that they agreed heat stress negatively impacted cattle performance, health, and welfare. The high level of agreement to these statements were also reflected in the thematic analysis (e.g., “concerned with animal welfare” and “…Cattle don’t want to eat or stay alive”). This demonstrates an understanding of and appreciation for the need to utilize strategies to reduce the negative impacts of heat stress on cattle. There is a plethora of research exploring how a variety of heat stress mitigation strategies can ameliorate heat stress conditions in dairy and beef cattle (reviewed by [1,17]). In recent years, the increase of extreme heat events has resulted in considerable cattle death loss in feedyards in the United States [22,23,24]. The NAHMS 2015 Death Loss Report [25] indicated that 9.3% of cattle death loss was attributed to weather-related issues by participants; although the specific weather issue was not reported, some portion of loss was likely due to extreme heat. In the current study, three-quarters of the survey respondents had experienced cattle death loss from extreme heat events. Although few respondents characterized their death loss as severe, any mortality due to the consequences of heat stress is a cattle welfare concern and can also have a significant financial impact. In the thematic analysis, one theme that was found in response to the question asking about how dealing with extreme heat events impacts employees was Morale; responses mentioned the emotional impact of working in conditions in which the welfare of the cattle is severely impacted, in addition to the physical stress of working in those conditions, which was also a theme that was identified. Finding solutions to managing these extreme events will benefit both the animals and the people caring for them.

Fewer respondents indicated that they strongly agreed that heat stress impacted carcass quality. Generally, when discussing the impacts of heat stress, carcass quality is not as often discussed (cattle performance is generally primary). If there was an improvement in carcass quality found when certain heat mitigation strategies were used, this would potentially incentivize more operators to implement these strategies. Past research has included carcass characteristics as outcomes of interest in studies exploring the benefits of some heat mitigation strategies (i.e., sprinklers and shade). The impact of shade provision on carcass outcomes has been the most studied. For example, Mitloehner et al. [29] reported a significant reduction in dark cutter prevalence in heifers that were shaded as compared to those that were not. In a retrospective analysis of historical dark cutting data from feedyards in Australia, Steel et al. [30] found that climactic conditions did play a role in the prevalence of dark cutting carcasses, although it was minimal. Interestingly, Steel et al. [30] speculated that one reason for the small impact of climatic conditions on dark cutting was that feedyards in Australia already have substantial systems in place to minimize the effects of heat stress on cattle; thus, in the United States, where adoption of certain mitigation strategies may be lower, climactic conditions may have a more considerable impact on the prevalence of dark cutting. The review paper by Edwards-Callaway et al. [17] includes a meta-analysis of published studies comparing shade vs. no shade conditions for feedyard cattle and found that cattle that were provided access to shade had greater HCWs, dressing percentages, marbling, and percentage of carcasses achieving at least Low Choice compared to those that did not have access to shade. In the thematic analysis in the current study, Cost was one theme identified in responses to a question asking respondents what resources would be helpful to managing heat stress (e.g., “Cost analysis to show the economic value of shade placement”). Perhaps synthesizing the results of studies that show a carcass value benefit to using heat abatement strategies would be beneficial to producers so that they could determine the economic benefits to adopting certain mitigation strategies; the return on investment of implementing a new management procedure is an important consideration for adoption.

There are many different approaches to managing heat stress that feedyards can implement [31]. Brown-Brandl [16] categorized heat stress management approaches into four different categories: feed, water, environmental influences, and handling. Most of the survey respondents indicated that they implemented or recommended the use of some type of heat mitigation strategy. Interestingly, while the majority of veterinarians and nutritionists recommended having a written protocol for describing heat stress mitigation strategies and monitoring, very few of the operators indicated that they had one in place. Some strategies may not require a written protocol; for example, if a feedyard utilizes permanent shade structures all year round, likely there are not procedural steps that warrant a formalized protocol, outside of a maintenance check. There are certainly other strategies (e.g., utilizing a sprinkler system or changing shipping times), many of which were utilized by operator respondents in this survey population, that would benefit from having a written protocol that indicated when to utilize, the procedural steps, and who was responsible. The U.S. Cattle Industry Feedyard Audit that is part of the National Cattlemen’s Beef Association (NCBA) Beef Quality Assurance (BQA) program includes audit criteria related to having a documented Inclement Weather protocol that addresses extreme conditions in addition to having records verifying that the protocol was implemented [32]. Although not feedyard-specific, studies exploring other important areas of animal care on livestock production facilities have reported a similarly low presence of written protocols (for disease treatment on dairies [33]; for downer cow management on dairies [34]). A 2004 study evaluated 56 feedyards using the BQA Feedyard Assessment document [35]; only one-third of the feedyards included in the study had documentation for the 18 required best management practices included in the assessment. Participants in that study mentioned that not having enough time to maintain and document protocols was a challenge [35]. Barnhardt et al. [35] also indicated that the smaller feedyards had fewer of the protocols. In studies on livestock operations where written protocols are scarce, there usually are informal protocols that are followed by caretakers [34,36], and feedyards could consider formalizing these into written documents that could be used in employee training programs.

Two of the most common heat mitigation strategies used or recommended by all three types of respondents was changing work hours and processing/shipping/re-implanting time. Additionally, most respondents indicated that they felt changing processing and shipping hours was effective at minimizing the effect of heat stress on cattle. Similarly, in a survey study of midwestern cattle feeders, Rusche et al. [20] indicated that all study participants reported avoiding handling cattle during heat events and two-thirds of respondents altered shipping schedules to avoid loading during the hottest times of the day. When cattle exert themselves physically, body temperature can rise, which would be detrimental to cattle during a heat stress event when their heat load is already high. The BQA program recommends avoiding or limiting handling cattle when the risk of heat stress is high [13]. Some types of processing may be easier to adjust than others; for example, shipping times are coordinated with slaughter plants so it is important to coordinate changes down the supply chain to avoid cattle waiting in trucks to unload at the plant in extreme weather if arrival times have not been adjusted.

Brown-Brandl et al. [37] explored wetting cattle during handling as a potential way to reduce heat load and reported some benefits in reducing body temperature, decreasing panting, and reducing recovery time. Studies have shown that using sprinklers to wet cattle can be effective at cooling cattle in both dairies and feedyards [38,39,40,41]. Slaughter plants that do not have covered facilities commonly use sprinklers as a way to cool cattle when environmental temperatures are high [42]. In this study, interestingly, the majority of veterinarians and nutritionists recommended using a sprinkler system or watering cattle down but the majority of operators did not utilize either strategy. Additionally, in the current study, using a sprinkler system had the lowest effectivity rating compared to other strategies across respondent roles. Interestingly, in the thematic analysis, when asked what they would do differently in face of another heat event, respondents mentioned utilizing water (e.g., “Sprinkle cattle more often” or “Cow cooling if with shade or water”). Rusche et al. [20] reported that 90% of respondents utilized a sprinkler system as a heat mitigation strategy, either wetting the cattle or the pen surface, but interestingly, only half of the respondents felt water application was successful. Rusche et al. [20] shared that some disadvantages to water application are the need to make daily application decisions based on the weather, managing mud that may be created when applying water, and the negative impacts of increased humidity on pen microclimates. Although details regarding why sprinklers were not used more often were not asked in the current study, as the whole beef supply chain is looking at ways to become more sustainable and minimize resource use [43], water use is likely a significant consideration when deciding whether or not to adopt sprinklers as a heat mitigation strategy. It would be important to evaluate mitigation strategies that utilize water through a lens of sustainability to identify trade-offs and synergies between the social, environmental, and economic components of feedyard operations.

Increasing water availability is another approach that was utilized by survey respondents; adding additional water was suggested most frequently by veterinarians and nutritionists and implemented less by operators. This strategy was ranked as moderately effective at minimizing the effects of heat stress. Rusche et al. [20] reported that one-third of the feedyard managers in their study felt additional water tanks during heat events was successful. In the current study, when respondents were asked what they would do differently in face of another heat event, Water and Feed management was the most common theme (e.g., “adequate water” or “Add water tanks”). Adequate water availability during extreme heat conditions is critical because cattle increase consumption when temperature increases, as it has a cooling effect [31]. There needs to be enough access to clean, and cool water if possible, so that cattle do not bunch and crowd around the available water, making a more detrimental microclimate. This can be done by reducing stocking density or adding additional water tubs. Research in dairy cattle has shown that cool/chilled water during times of heat stress is effective at reducing body temperature and respiration rates [44,45], emphasizing the important role of water temperature in heat stress mitigation.

Modification of feeding strategies both before, during, and after an extreme heat event was also identified as a highly utilized and recommended strategy by the majority of respondents in the current study. Water and Feed Management was also the most common theme found in the thematic analysis, particularly in response to the question asking respondents what they would do differently in the face of another extreme heat event. Changing feed delivery times was one strategy used and recommended by a large proportion of survey respondents, although the mean effectivity rating was slightly lower than some other strategies. By delivering feed later in the day, the peak metabolic heat load of the cattle would coincide with the cooler evening temperatures, thus facilitating heat dissipation [46,47]. Barajas et al. [48] reported positive impacts on cattle performance when adjusting feed delivery to later in the day. Rusche et al. [20] reported that less than a quarter of respondents indicated that they felt feed additives (e.g., essential oils and Hydro-Lac, Form-A-Feed, Inc., Minnesota, USA) were successful heat stress mitigation strategies. There are many different management adaptations related to nutrition that can be altered before, during, and after heat stress events, and this area should be further explored to understand successes and challenges experienced by those who use these strategies to mitigate heat stress in feedyard cattle. In the thematic analysis, Research and Data was found to be a common identified need in this area, suggesting that there is opportunity to evaluate additional strategies.

The use of shade was reported as a percentage of respondents that had shade in some pens due to sample size and a large range of proportion of pens that were shaded in participating feedyards. Approximately half of the respondents had shade in at least some home pens and some holding pens. Nearly three-quarters had shade in some hospital pens. There is little published information outside of a few regional surveys reporting the presence of shade in feedyards across the United States. Two survey studies, one with feedyard managers [18] and one with consulting nutritionists [19], reported less than one-fifth of respondents provided shade structures in pens. A more recent study of cattle feeders [20] indicated that just over half of the respondents utilized shade, and a similar proportion of them felt it was a successful heat-mitigation strategy. Similarly, in the current study, the majority of respondents indicated that they felt shade was effective at minimizing the effect of heat stress on cattle; in the thematic analysis, approximately one-third of those who answered mentioned shade utilization when asked what they would do differently in the face of another extreme heat event. There is substantial evidence that shade provision can positively affect cattle performance and welfare (reviewed by Edwards-Callaway et al. [17]). It should be noted that not all shade is effective and if poorly designed could have detrimental effects on cattle welfare and performance; shade should be high enough to allow for substantial air movement, it should be oriented to maximize coverage, and there should be enough shade to accommodate animal need [17]. The industry would benefit from an economic analysis of shade implementation in feedyards, as the last published economic assessment was from 2003 [26] and the landscape is considerably different two decades later [17]. Shade requires a considerable capital investment, and thus it is imperative to understand the return on investment.

Study respondents utilized a variety of different monitoring tools and indicators to identify when to implement their mitigation strategies. Cattle behaviors, daily forecast, and multiple day heat event anticipated were the most highly selected indicators used to determine when to implement heat stress mitigation strategies. Local forecast, heat index, and temperature humidity index were the most highly selected tools utilized for heat event monitoring. Respondents mainly utilized multiple indicators or tools; however, this study was unable to provide the level of detail on how these types of indicators and monitoring tools could be improved or what other resources would be valuable. Rusche et al. [20] reported similar “mitigation strategy preparation triggers” in their survey study, although the lists that each study provided were slightly different. In Rusche et al. [20], the majority of study participants relied on calendar schedules and weather conditions. Being able to predict and forecast heat stress risks enables feedyard operators and their support team to effectively prepare to ultimately minimize any negative impacts on cattle [2].

Interestingly, the agreement statements regarding how helpful the involvement of a veterinarian or a nutritionist was in making heat stress management decisions had variable results. The operators and nutritionists selected moderate agreement with the statements while the veterinarians agreed more strongly that involving both nutritionists and veterinarians would be helpful. The 2011 NAHMS Feedlot study reported that nearly all feedyards with a capacity greater than 1000 cattle (96.6%) used the services of a veterinarian in some capacity to assist with ensuring cattle health [21]. Similarly, the vast majority of feedyards with a capacity greater than 1000 cattle (95.2%) in the 2011 NAHMS Feedlot study also reported using the services of a nutritionist [21]. In the current study, a majority of operators did answer yes when asked if they consult with their veterinarian (n = 15, 68.2%) or nutritionist (n = 17, 77.3%) to determine heat stress mitigation strategies. Although feedyards enlist the services of these experts, perhaps assisting with heat stress management is not considered one of the primary responsibilities of nutritionists and veterinarians. Both of these stakeholders can provide valuable advice when considering potential strategies to implement, in addition to dealing with any negative cattle health and welfare impacts that may result from an extreme heat event. Feedyard operators should consider increasing the involvement of their veterinarians and nutritionists in developing strategies, writing protocols, monitoring animal outcomes, and conducting trainings with employees. Although not related to heat stress mitigation, other studies have indicated that veterinarians are often responsible for training employees [49], are interested in participating in more employee training [50,51], and have had success organizing training workshops to teach producers about important welfare topics [52].

## 5. Conclusions

These results indicate a variety of strategies are being used by cattle feedyard operators, veterinarians, and nutritionists to mitigate heat stress. However, opportunities remain to improve resources for forecasting heat stress events, improving mitigation strategies and economic assessments of their effectiveness, and improving strategies that consider mitigating the negative effects of heat stress on workers and cattle alike. While nutritionists and veterinarians are viewed as trusted sources of information by feedyard operators, there is room to improve knowledge transfer on the issue of managing heat loads in cattle from these experts to operators and vice versa. Future research should focus on evaluating how different heat stress mitigation strategies can contribute to the overall sustainability of feedyard production systems; while some strategies may improve the welfare of animals (e.g., improved gain, reduced mortality losses), they may use more or fewer resources (e.g., water use for sprinklers to cool the cattle or the ground) from beef systems.

## Figures and Tables

**Table 1:** Survey respondent demographics (n = 56).

Demographic	n, %
Role Feedyard Owner/Operator	22, 39.3%
Nutritionist	8, 14.3%
Veterinarian	26, 46.4%
Gender	
Man	46, 82.1%
Woman	8, 14.3%
Other	0, 0%
Prefer not to answer	1, 1.8%
No answer	1, 1.8%
Race and ethnicity	
American Indian or Alaska Native	1, 1.8%
Asian	1, 1.8%
Black or African American	0,0%
Native Hawaiian or Other Pacific Islander	0, 0%
White	48, 85.7%
Hispanic, Latino/a/x, Spanish	0, 0%
Hispanic, Latino/a/x, Spanish/White	1, 1.8%
Other	0, 0%
Prefer not to answer	3, 5.4%
No answer	2, 3.6%
Age	
20–29	3, 5.4%
30–39	7, 12.5%
40–49	10, 17.9%
50–59	14, 25%
60–69	5, 8.9%
70+	7, 12.5%
No answer	10, 17.9%
Region ^1^	
Midwest	36, 64.3%
West	17, 30.4%
Southwest	15, 26.8%
Southeast	0, 0.0%
Northeast	0, 0%
Alaska or Hawaii	0, 0%
Canada	1, 1.8%
No Answer	3, 5.4%

^1^ Region: Midwest (ND, SD, NE, KS, MN, IA, MO, WI, IL, IN, MI, OH), West (CA, OR, WA, NV, ID, MT, WY, UT, CO), Southwest (AZ, NM, OK, TX), Southeast (AK, LA, MS, AL, GA, TN, KY, WV, VA, NC, SC, FL), Northeast (MD, DE, PA, NJ, CT, NY, RI, MA, NH, VT, ME).

**Table 2:** Summary of cattle demographics reported by operators (n = 22). Respondents were able to select more than one answer for breed and hide color, so totals may be greater than 100%.

Demographic	Category	n, %
Breed	Brahman (bos indicus) influence	8, 36.4%
	British	18, 81.8%
	Continental	12, 54.5%
	British continental-cross	13, 59.1%
	Holstein	6, 27.3%
	Beef on dairy	10, 45.5%
	Mexican	5, 22.7%
	Other	2, 9.1%
	No answer	1, 4.5%
Hide Color	Black	22, 100%
	Red	19, 86.4%
	Gray	13, 59.1%
	Yellow	13, 59.1%
	Other	3, 13.6%
Sex class	Heifers only	0, 0%
	Steers only	1, 4.5%
	Both Heifers and Steers	21, 95.5%
	No answer	0, 0.0%
Average feedyard capacity	<1000	4, 18.2%
(number of animals)	1000 or more	14, 63.6%
	1000–7999	3, 13.6%
	8000–15,999	0, 0%
	16,000–31,999	3, 13.6%
	32,000 or more	8, 36.4%
	No answer	4, 18.2%

**Table 3:** Summary of respondent answers by role (operators, n = 22; veterinarians, n = 26; nutritionists, n = 8) when asked if they utilize or recommend the listed heat stress mitigation strategies.

Survey Question— For Extreme Heat Events Do You (or Recommend to):	Role (n, %)		
	Operator	Veterinarian	Nutritionist
add additional water availability?			
Yes	8, 36.4%	23, 88.5%	7, 87.5%
No	14, 63.6%	3, 11.5%	1, 12.5%
No answer	0, 0.0%	0, 0.0%	0, 0.0%
change work hours?			
Yes	16, 72.7%	26, 100%	8, 100%
No	6, 27.3%	0, 0.0%	0, 0.0%
No answer	0, 0.0%	0, 0.0%	0, 0.0%
change processing/re-implanting/shipping times?			
Yes	20, 91%	25, 96.2%	8, 100%
No	1, 4.5%	1, 3.8%	0, 0.0%
No answer	1, 4.5%	0, 0.0%	0, 0.0%
utilize a sprinkler system?			
Yes	5, 22.7%	16, 61.5%	6, 75%
No	16, 72.7%	10, 38.5%	2, 25%
No answer	1, 4.5%	0, 0.0%	0, 0%
water cattle down?			
Yes	5, 22.7%	12, 46.2%	5, 62.5%
No	16, 72.7%	13, 50%	3, 37.5%
No answer	1, 4.5%	1, 3.8%	0, 0%
provide bedding?			
Yes	10, 45.5%	22, 84.6%	6, 75%
No	11, 50%	4, 15.4%	2, 25%
No answer	1, 4.5%	0, 0.0%	0, 0%
modify feeding strategies during?			
Yes	12, 54.5%	20, 76.9%	8, 100%
No	10, 45.5%	6, 23.1%	0, 0%
No answer	0, 0.0%	0, 0.0%	0, 0%
modify feeding strategies after?			
Yes	16, 72.7%	17, 65.4%	5, 62.5%
No	6, 27.3%	9, 34.6%	3, 37.5%
No answer	0, 0.0%	0, 0.0%	0, 0%

**Table 4:** Summary of respondents by role (operators, n = 22; veterinarians, n = 26; nutritionists, n = 8) in response to questions related to indicators used to implement heat stress mitigation strategies and monitoring tools used.

	Role (n, %)		
Survey Question	Operator	Veterinarian	Nutritionist
Which indicators do you use to determine the need for implementing heat stress mitigation strategies?			
Cattle behaviors	17, 77.3%	22, 84.6%	5, 62.5%
Daily forecast	19, 86.4%	20, 76.9%	6, 75%
Historical weather data	2, 9.1%	8, 30.8%	4, 50%
Multiple day heat event anticipated	18, 81.8%	23, 88.5%	7, 87.5%
Protocols	4, 18.2%	11, 42.3%	1, 12.5%
I don’t use any tools	1, 4.5%	0, 0.0%	0, 0.0%
Other	0, 0.0%	1, 3.8%	1, 12.5%
No answer	0, 0.0%	0, 0.0%	0, 0.0%
What heat event monitoring tools do you use?			
Cattle comfort index	3, 13.6%	13, 50%	2, 35%
Heat index	13, 59.1%	16, 61.5%	4, 50%
Local forecast	19, 86.4%	15, 57.7%	2, 35%
Temperature humidity index (THI)	12, 54.5%	19, 73.1%	6, 75%
Weather station on premises	5, 22.7%	7, 26.9%	3, 37.5%
I don’t use any tools	1, 4.5%	0, 0.0%	1, 12.5%
Other	0, 0.0%	0, 0.0%	1, 12.5%
No answer	0, 0.0%	0, 0.0%	0, 0.0%

**Table 5:** Respondents were asked to select how effective they felt a list of heat stress mitigation strategies were on a scale of 0 (not effective) to 10 (extremely effective). The 0 to 10 scale was divided into three categories of effectivity (Low = 0 to 3; Middle = 4 to 7; High = 8 to 10) and the number of respondents within each category were calculated. There was a total of 22 operators, 26 veterinarians, and 8 nutritionists included in the analysis.

		Effectiveness Category (n)			
	Mean ± SD	Low	Middle	High	No Answer
**Survey Statement—** Please rate the following on a scale from “not effective” (0) to “extremely effective” (10) as it relates to minimizing the effects of heat stress on cattle.					
Changing water availability					
Operator	7 ± 3.2	3	5	8	6
Veterinarian	7.8 ± 2.3	1	9	16	0
Nutritionist	7.7 ± 1.8	0	4	3	1
Changing feeding strategies					
Operator	5.9 ± 2.9	4	6	5	7
Veterinarian	6.6 ± 2.5	2	12	10	2
Nutritionist	6 ± 3.7	3	2	3	0
Providing shade					
Operator	7.1 ± 3.1	4	4	11	3
Veterinarian	8.7 ± 1.2	0	2	21	3
Nutritionist	8.6 ± 1.9	0	2	5	1
Changing processing and shipping hours					
Operator	8.0 ± 2.2	1	7	14	0
Veterinarian	8.6 ± 1.4	0	4	21	1
Nutritionist	8.8 ± 1.7	0	1	7	0
Using a sprinkler system					
Operator	5.2 ± 3.3	5	6	4	7
Veterinarian	6.1 ± 2.9	4	10	7	5
Nutritionist	5.5 ± 2.9	2	3	1	2

**Table 6:** Respondents were asked to select their level of agreement on a scale of 0 (strongly disagree) to 10 (strongly agree) with statements related to the resources used in relation to heat stress management. The 0 to 10 scale was divided into three categories of agreement (Low = 0 to 3; Middle = 4 to 7; High = 8 to 10) and the number of respondents within each category were calculated. There was a total of 22 operators, 26 veterinarians, and 8 nutritionists included in the analysis.

		Agreement Category			
Survey Statement	Mean ± SD	Low	Middle	High	No answer
The triggers I use to begin implementing heat stress mitigation work					
Operator	6.7 ± 2.6	3	8	8	3
Veterinarian	6.6 ± 1.9	1	17	7	1
Nutritionist	6.3 ± 2.4	1	3	3	1
The heat stress monitoring tools I use are helpful					
Operator	7 ± 2.4	3	7	11	1
Veterinarian	7.3 ± 1.9	3	9	13	1
Nutritionist	7.1 ± 1.6	0	4	4	0
Involving a veterinarian in heat stress management decisions is helpful					
Operator	5.9 ± 3.7	5	4	8	5
Veterinarian	8.3 ± 1.6	0	8	18	0
Nutritionist	5.0 ± 1.5	1	6	0	1
Involving a nutritionist in heat stress management decisions is helpful					
Operator	6.2 ± 3.6	5	4	10	3
Veterinarian	8.0 ± 2.1	0	9	16	1
Nutritionist	7.5 ± 2.3	0	4	4	0

**Table 7:** Respondents were asked to select their level of agreement on a scale of 0 (strongly disagree) to 10 (strongly agree) with statements related to the impact that heat stress has on various cattle attributes. The 0 to 10 scale was divided into three categories of agreement (Low = 0 to 3; Middle = 4 to 7; High = 8 to 10) and the number of respondents within each category were calculated. There was a total of 22 operators, 26 veterinarians, and 8 nutritionists included in the analysis.

		Agreement Category (n)			
Survey Statement	Mean ± SD	Low	Middle	High	No Answer
Heat stress negatively impacts cattle performance					
Operator	9.1 ± 1.8	1	1	20	0
Veterinarian	9.2 ± 1.1	0	3	23	0
Nutritionist	9.8 ± 0.7	0	0	8	0
Heat stress negatively impacts cattle health					
Operator	8.1 ± 2.2	1	5	16	0
Veterinarian	8.6 ± 1.9	1	6	19	0
Nutritionist	9.8 ± 0.7	0	0	8	0
Heat stress negatively impacts cattle welfare					
Operator	7.8 ± 2.6	1	7	14	0
Veterinarian	8.9 ± 1.7	1	5	20	0
Nutritionist	10.0 ± 0	0	0	8	0
Heat stress negatively impacts carcass quality					
Operator	5.8 ± 2.9	5	7	7	3
Veterinarian	7.3 ± 2.3	1	13	10	2
Nutritionist	7.3 ± 2.3	0	4	4	0

**Table 8:** Themes, definitions, and examples of responses for all themes identified within the thematic analysis. The questions ^1^ that each theme was found in is also noted.

Themes	Definition	Question	Primary Examples
Water and Feed Management	References to changing feed rations, introducing feed additives, altering feed times, or adding additional water.	Q1, Q2	“Feed additives to reduce heat stress impact are vastly underused” “reduce morning feed delivery”
In Pen Infrastructure	Structure within the pen such as shade, sprinklers, bedding, or air flow	Q1, Q2, Q3	“More shade and water availability, utilize bedding” “Improve air flow is critical if possible”
Cattle Characteristics	Comments on cattle that are perceived to increase heat stress such as breed, hide-color, weight, EPDs or genetics.	Q1, Q2, Q3	“Shade in high risk cattle (those that are fat, close to marketing, have had health issues, black-hided)…” “cattle breed and body type make a huge difference on tolerance to heat stress”
Cattle Movement Strategies	Statements about time of movement (e.g., processing) or moving to pastures during heat events.	Q1, Q3	“move cattle out of pens to adjacent grasslands” “Work cattle early”
Preparation	Starting mitigation earlier such as weather tracking or heat mitigation strategies.	Q1, Q3	“recommend a strategy of seasonal heat abatement not event” “start mitigation earlier before the dangerous heat index becomes a reality”
Research and Data	Statements showing interest in more research and data relative to heat stress events or mitigation.	Q1, Q2	“more research in this and improving ventilation in housed feedlot cattle is greatly needed” “Data from well managed trials”
Cost	Relative to cost of labor or infrastructure.	Q2, Q3	“labor availability” “Cost analysis to show the economic value of shade placement.”
Moral	Any emotional impact heat events or stress has on workers.	Q3	“It sucks. It’s hot. No one wants to work. Cattle don’t want to eat or stay alive.” “Emotional impact, workload, financial loss, public perception”
Physical Stress on Employees	Any physical impact heat events or stress has on workers.	Q3	“Them having heat stress just like the animals” “hard on staff working in heat”
Monitoring Resources	Use or availability of heat monitoring tools.	Q2	“We need better tools to predict heat stress. To me it’s more about how drastic the change is and how long cattle have had to acclimate.” “An easy to use heat stress dashboard that predicts heat stress (THI) events.”
Other		Q1, Q2, Q3	“Pray for wind. That rarely happens” “No impact”

^1^ Survey questions asked: Q1—Please share what you would do differently in the face of another extreme heat event.; Q2—What resources would be helpful to you in regard to managing heat stress? Please explain.; Q3—How does dealing with an extreme heat event impact you and/or your employees? What are the most difficult aspects of dealing with an extreme heat event? Please explain.

## Data Availability

Data available upon request to the corresponding author.

## Supplementary Materials

The following supporting information can be downloaded at: https://www.mdpi.com/article/10.3390/ani13193029/s1, Document S1: Survey Questions.
