# Supplementary material for: Heat Stress Mitigation Strategies in Feedyards: Use, Perceptions, and Experiences of Industry Stakeholders
Source: Animals (Basel). 2023 Sep 26;13(19):3029. doi: 10.3390/ani13193029 (PMC10572074; doi:10.3390/ani13193029)
Supplement: Supplementary file 1 [file animals-13-03029-s001.zip › animals-2608015-supplementary.pdf]

Q1 Select your role in the feedlot industry (select all that apply):

- ○ Feedlot operator (owner or manager) (1)
- ○ Veterinarian (2)
- ○ Nutritionist (3)
- ○ Other, please specify. (4) ____

End of Block: Default Question Block

Start of Block: Block 1

Producer Track

FACILITY AND CATTLE DESCRIPTION

Q2 Please complete the following for the feedlot that you operate:

- ▢ Location(state) (1) ____
- ▢ Zip Code (2) ____
- ▢ One time capacity (3) ____
- ▢ Average number of cattle per pen (4) ____
- ▢ Average pen density (square feet/head) (5) ____

Q3 What are your cattle demographics reported as a percentage of your one-time capacity?

Q4 Sex class (%):

- ☐ Steers (1) ____
- ☐ Heifers (2) ____

Q5 Breed type (%):

- ☐ Brahman (bos indicus) influence (1) ____
- ☐ British (2) ____
- ☐ Continental (3) ____
- ☐ British continental-cross (4) ____
- ☐ Holstein (5) ____
- ☐ Beef on dairy (6) ____
- ☐ Mexican (7) ____
- ☐ Other, please specify (8) ____

Q6 Hide color (%):

- [ ] Black (1) ____
- [ ] Red (2) ____
- [ ] Gray (3) ____
- [ ] Yellow (4) ____
- [ ] Other, please specify (5) ____

Q7 What is the average incoming weight (lbs) for the following groups:

- [ ] Calves (1) ____
- [ ] Backgrounders (2) ____
- [ ] Yearlings (3) ____

Q8 What are the average days on feed for the following groups:

- ☐ Calves (1) ____
- ☐ Backgrounders (2) ____
- ☐ Yearlings (3) ____
- ☐ Holstein (4) ____
- ☐ Beef on dairy (5) ____
- ☐ Wagyu (6) ____
- ☐ Other type of cattle, please specify (7) ___________________________

Q9 What is your target finish weight (lbs) for the following groups:

- ☐ Calves (1) ____
- ☐ Backgrounders (2) ____
- ☐ Yearlings (3) ____
- ☐ Holstein (4) ____
- ☐ Beef on dairy (5) ____
- ☐ Wagyu (6) ____
- ☐ Other (7) ____

Q10 What feedstuffs do you typically incorporate into your rations? (select all that apply)

- ☐ Alfalfa (1)
- ☐ Barley (2)
- ☐ Co-products (3)
- ☐ Corn (4)
- ☐ Fat/Oil (5)
- ☐ Hay/Straw (6)
- ☐ Milo (7)
- ☐ Sorghum (8)
- ☐ Wheat (9)
- ☐ Other, please specify (10) ____

End of Block: Block 1

Start of Block: Block 2

HEAT STRESS MITIGATION STRATEGIES-GENERAL

Q11 Do you implement heat stress mitigation strategies at your feedlot?

- ○ Yes (1)
- ○ No (2)

Q12 Do you implement additional and/or different heat stress mitigation strategies for sick cattle?

- ○ Yes (1)
- ○ No (2)

Q13 Do you have a written protocol describing heat stress mitigation strategies and monitoring?

- ○ Yes (1)
- ○ No (2)

Q14 Do you target certain classes of cattle during an extreme heat event or do you apply the same strategies throughout your feedlot?

- ○ Target certain classes (3)
- ○ Same strategies throughout (4)

Q15 Do you consult with your veterinarian to determine heat stress mitigation strategies?

- ○ Yes (1)
- ○ No (2)

Q16 Do you consult with your nutritionist to determine heat stress mitigation strategies?

- ○ Yes (1)
- ○ No (2)

Q17 Which indicators do you use to determine the need for implementing heat stress mitigation strategies? Select all that apply.

- ☐ Cattle behavior (1)
- ☐ Daily forecast (2)
- ☐ Historical weather data (3)
- ☐ Multiple day heat event anticipated (4)
- ☐ Protocols (5)
- ☐ I don't use any tools (6)
- ☐ Other (7) ____

Q18 What heat event monitoring tools do you use? Select all that apply.

- ☐ Cattle comfort index (1)
- ☐ Heat index (2)
- ☐ Local forecast (3)
- ☐ Temperature humidity index (THI) (4)
- ☐ Weather station on premises (5)
- ☐ I don't use any tools (6)
- ☐ Other (7) ____

Q19 Are you Beef Quality Assurance (BQA) certified?

- ○ Yes (1)
- ○ No (2)

Q20 Are the majority of your employees Beef Quality Assurance (BQA) certified?

- ○ Yes (1)
- ○ No (2)

End of Block: Block 2

Start of Block: Block 3

WATER

Q21 What is your average linear availability per head for water?

____

Q22 What is the location of the waterer(s) within the pens?

- ▢ Fence-line (1)
- ▢ Center (2)
- ▢ Other, where? (3) ____

Q23 How many waterers do you have per pen, on average?

____

Q24 Do you add additional water availability during extreme heat events?

- ○ Yes (1)
- ○ No (2)

Q25 Do you monitor water temperature?

- ○ Yes (1)
- ○ No (2)

End of Block: Block 3

Start of Block: Block 4

MANAGEMENT

Q26 Do you change work hours during extreme heat events?

- ○ Yes (1)
- ○ No (2)

Q27 Do you change processing/re-implanting/shipping times during extreme heat events?

- Yes (1)
- No (2)

Q28 What is the latest time of day or highest temperature that you recommend working cattle during extreme heat events?

____

End of Block: Block 4

Start of Block: Block 5

PEN MANAGEMENT

Q29 Do you utilize a sprinkler system for heat stress mitigation?

- Yes (1)
- No (2)

Q30 Do you water cattle down during extreme heat events?

- Yes (1)
- No (2)

Q31 Do you provide bedding during extreme heat events?

- Yes (1)
- No (2)

End of Block: Block 5

Start of Block: Block 6

PEN CONSTRUCTION

Q32 Please select which best describes your pens:

- Inside (1)
- Outside (2)
- Both (3)

Q33 Are your pens designed with earthen mounds in the center of the pen?

- Yes (1)
- No (2)

Q34 What type of fencing do you have between pens? Select all that apply.

- Open/cable wire (1)
- Solid windbreak (2)
- Other (3) ____

Q35 What percentage of your home pens have shade? ________________________________

Q36 What percentage of your hospital pens have shade? ________________________________

Q37 What percentage of your holding pens (e.g., processing and shipping) have shade?
____

Q38 If you have shade in any of the above pens, please describe the following for your shade structures:

▢ Material (1) ____

▢ Height (2) ____

▢ Sq/ft/animal (3) ____

Q39 Do you utilize windbreaks?

○ Yes (1)

○ No (2)

Display This Question:

If Do you utilize windbreaks? = Yes

Q39.2 Are your windbreaks removed during summer?

○ Yes (1)

○ No (2)

End of Block: Block 6

Start of Block: Block 7

NUTRITION

Q40 Do you modify feeding strategies during extreme heat events?

○ Yes (1)

○ No (2)

Display This Question:

If Do you modify feeding strategies during extreme heat events? = Yes

Q40.2 If you modify feeding strategies during extreme heat events please answer yes or no:

☐ Do you alter feed composition? (1)

☐ Do you change feed delivery times? (2)

☐ Do you utilize different feed additives during heat stress events? (3)

Q41 Do you modify feeding strategies after extreme heat events to help cattle recover?

○ Yes (1)

○ No (2)

Display This Question:

If Do you modify feeding strategies after extreme heat events to help cattle recover? = Yes

Q41.2 If you modify feeding strategies after extreme heat events to help cattle recover please answer yes or no:

☐ Do you alter feed composition? (1)

☐ Do you change feed delivery times? (2)

☐ Do you utilize different feed additives during heat stress events? (3)

End of Block: Block 7

Start of Block: Block 8

PERCEPTIONS

Q42 Please rate the following on a scale from "not effective" (0) to "extremely effective" (10) as it relates to minimizing the effects of heat stress on cattle. Please check not applicable if you do not know.

Not Applicable

	0	1	2	3	4	5	6	7	8	9	10
Changing water availability ()											
Changing feeding strategies ()											
Providing shade ()											
Changing processing and shipping hours ()											
Using a sprinkler system ()											

Q43 Please rate the following on a scale from "strongly disagree" (0) to "strongly agree" (10). Please check not applicable if you do not know.

Not Applicable

0 1 2 3 4 5 6 7 8 9 10

Heat stress negatively impacts cattle performance. ()	
Heat stress negatively impacts cattle health. ()	
Heat stress negatively impacts cattle welfare. ()	
Heat stress negatively impacts carcass quality. ()	
The triggers I use to begin implementing heat stress mitigation work. ()	
The heat stress monitoring tools I use are helpful. ()	
Involving a veterinarian in heat stress management decisions is helpful. ()	
Involving a nutritionist in heat stress management decisions is helpful. ()	

Q44 Are there any additional comments you would like to provide.

____

Q45 What are some obstacles in implementing heat stress mitigation strategies? Select all that apply.

- [ ] Cost (1)
- [ ] Expertise (2)
- [ ] Facility limitations (3)
- [ ] Labor (4)
- [ ] Natural resources (e.g., water) (5)
- [ ] Time (6)
- [ ] Training (7)
- [ ] Scheduling (8)
- [ ] Lack of data to demonstrate effectiveness (9)
- [ ] Other, please explain. (10) ____

Q46 What resources would be helpful to you in regards to managing heat stress? Please explain.

____

Q47 How does dealing with an extreme heat event impact you and/or your employees? What are the most difficult aspects of dealing with an extreme heat event? Please explain.

____

End of Block: Block 8

Start of Block: Block 9

EXTREME HEAT EVENTS

Q48 Have you experienced cattle death loss from extreme heat events?

- Yes (1)
- No (2)

Display This Question:

If Have you experienced cattle death loss from extreme heat events? = Yes

Q48.2 How frequently do you experience cattle death loss from extreme heat events?

- Multiple times per year (1)
- Once per year (2)
- Every few years (3)

Display This Question:

If Have you experienced cattle death loss from extreme heat events? = Yes

Q48.3 During which months of the year have you experienced extreme heat events?

- ☐ April (4)
- ☐ May (5)
- ☐ June (6)
- ☐ July (7)
- ☐ August (8)
- ☐ September (9)
- ☐ Other (12) ____

Display This Question:

If Have you experienced cattle death loss from extreme heat events? = Yes

Q48.4 Would you consider your loss as:

- ○ Minor (5)
- ○ Moderate (6)
- ○ Severe (7)

Display This Question:

If Would you consider your loss as: = Minor

Or Would you consider your loss as: = Moderate

Or Would you consider your loss as: = Severe

Q48.4.2 Please explain your selection (minor, moderate or sever) for your loss during extreme heat events (e.g, amount of cattle lost, impact on employees)

____

Q49 What do you consider the class(es) of animals with the highest risk for death loss?

____

Q50 Please share what you would do differently in the face of another extreme heat event.

____

End of Block: Block 9

Start of Block: Block 10

DEMOGRAPHICS

Q51 What gender do you identify with?

- ○ Male (1)
- ○ Female (2)
- ○ Other (3)
- ○ Prefer not to answer (4)

Q52 How would you best describe yourself? Select all that apply.

- ▢ American Indian or Alaska Native (1)
- ▢ Asian (2)
- ▢ Black or African American (3)
- ▢ Native Hawaiian or Other Pacific Islander (4)
- ▢ White (5)
- ▢ Other, please specify: (6) ____
- ▢ Prefer not to answer (7)

Q53 Do you identify as Hispanic, Latino/a/x or Spanish origin?

- ○ Yes (1)
- ○ No (2)
- ○ Other, please specify: (3) ____
- ○ Prefer not to answer (4)

Q54 What is your age (in years)?

____

Q55 Approximately how many years have you been working in the cattle industry?

◯ Less than 1 year (1)

◯ 1 to 5 years (2)

◯ 5 to 10 years (3)

◯ >10 years (4)

End of Block: Block 10

Veterinarian and Nutritionist Track

Q2 How many feedlots do you oversee?

◯ 1 (1)

◯ +1 (Please enter how many) (2)
____

End of Block: Block 19

Start of Block: VET/NUT 1

FACILITY AND CATTLE DESCRIPTION

Q3 Please complete the following for the feedlot that you operate/consult at:

▢ Location(state) (1)
____

▢ Zip Code (2) ____

Q4 Breed type fed at the feedlot. Select all that apply.

- ☐ Brahman (bos indicus) influence (1)
- ☐ British (2)
- ☐ Continental (3)
- ☐ British continental-cross (4)
- ☐ Holstein (5)
- ☐ Beef on dairy (6)
- ☐ Mexican (7)
- ☐ Other (8)

Q5 Recommended feedstuffs incorporated into feedlot rations. Select all that apply.

- ▢ Alfalfa (1)
- ▢ Barley (2)
- ▢ Co-products (3)
- ▢ Corn (4)
- ▢ Fat/Oil (5)
- ▢ Hay/Straw (6)
- ▢ Milo (7)
- ▢ Sorghum (8)
- ▢ Wheat (9)
- ▢ Other, please specify (10) ____

End of Block: VET/NUT 1

Start of Block: Vet 1.5

Display This Question:

If Select your role in the feedlot industry (select all that apply): = Veterinarian

Q6 Do you have a Veterinary/Client/Patient Relationship (VCPR)?

- ○ Yes (1)
- ○ No (2)

End of Block: Vet 1.5

Start of Block: VET/NUT 2

HEAT STRESS MITIGATION STRATEGIES-GENERAL

Q7 Do you have a recommended written protocol describing heat stress mitigation strategies and monitoring you provide to the feedlot?

- ○ Yes (1)
- ○ No (2)

Q8 Do you recommend targeting certain classes of cattle during extreme heat events or do you apply the same strategies throughout the feedlot?

- ○ Target certain classes (3)
- ○ Same strategies (4)

Q9 Which indicators do you recommend to use to determine the need for implementing heat stress mitigation strategies? Select all that apply.

- ▢ Cattle behavior (1)
- ▢ Daily forecast (2)
- ▢ Historical weather data (3)
- ▢ Multiple day heat event anticipated (4)
- ▢ Protocols (5)
- ▢ I don't recommend any tools (6)
- ▢ Other (7) ____

Q10 What heat event monitoring tools do you recommend to use? Select all that apply.

- ☐ Cattle comfort index (1)
- ☐ Heat index (2)
- ☐ Local forecast (3)
- ☐ Temperature humidity index (THI) (4)
- ☐ Weather station on premises (5)
- ☐ I don't recommend any tools (6)
- ☐ Other (7) ____

Q11 Does the feedlot you consult with implement heat stress mitigation strategies?

- ○ Yes (3)
- ○ No (4)
- ○ I do not know (5)

Display This Question:

If Does the feedlot you consult with implement heat stress mitigation strategies? = Yes

Q11.2 Does the feedlot that you consult with implement additional and/or different heat stress mitigation strategies for sick cattle?

- ◯ Yes (1)
- ◯ No (2)
- ◯ I do not know (3)

Q12 Does the feedlot you oversee consult with you during extreme heat events?

- ◯ Yes (1)
- ◯ No (2)

Q13 Are you Beef Quality Assurance (BQA) certified?

- ◯ Yes (1)
- ◯ No (2)

End of Block: VET/NUT 2

Start of Block: VET/NUT 3

WATER

Q14 What is your recommended average linear availability per head for water?

____

Q15 Do you recommend adding additional water availability during extreme heat events?

◯ Yes (3)

◯ No (4)

Q16 Do you recommend monitoring water temperature?

◯ Yes (1)

◯ No (2)

End of Block: VET/NUT 3

Start of Block: VET/NUT 4

MANAGEMENT

Q17 Do you recommend changing work hours during extreme heat events?

◯ Yes (1)

◯ No (2)

Q18 Do you recommend changing processing/re-implanting/shipping times during extreme heat events?

◯ Yes (1)

◯ No (2)

Q19 What is the latest time of day or temperature that you recommend working cattle during extreme heat events?

____

End of Block: VET/NUT 4

Start of Block: VET/NUT 5

PEN MANAGEMENT

Q20 Do you recommend utilizing a sprinkler system for heat stress mitigation?

- Yes (1)
- No (2)

Q21 Do you recommend watering cattle down during extreme heat events?

- Yes (1)
- No (2)

Q22 Do you recommend providing bedding during extreme heat events?

- Yes (1)
- No (2)

End of Block: VET/NUT 5

Start of Block: Vet/Nut 6

PEN CONSTRUCTION

Q23 Please select which best describes the pens at the feedlot you consult with:

- Inside (1)
- Outside (2)
- Both (3)
- I do not know (4)

Q24 Are the pens at the feedlot you consult with designed with earthen mounds in the center of the pen?

- Yes (1)
- No (2)
- I do not know (3)

Q25 What type of fencing does the feedlot have between pens? (Select all that apply)

- Open/Cable Wire (1)
- Solid windbreak (2)
- Other (Please explain) (3) ____
- I do not know (4)

Q26 What percentage of the feedlot home pens have shade?

____

Q27 What percentage of the feedlot hospital pens have shade?

____

Q28 What percentage of the feedlot holding pens (e.g., processing and shipping) have shade?

____

Q29 If the feedlot has shade in any of the above pens, please describe the following:

- ▢ Material (1) ____
- ▢ Height (2) ____
- ▢ Sq/Ft/Animal (3) ____
- ▢ I do not know any of the above answers (4)

Q30 Does the feedlot you consult with utilize windbreaks?

- ◯ Yes (1)
- ◯ No (2)
- ◯ I do not know (3)

Display This Question:

If Does the feedlot you consult with utilize windbreaks? = Yes

Q30.2 Are the windbreaks removed during summer?

- ◯ Yes (1)
- ◯ No (2)
- ◯ I don't know (3)

End of Block: Vet/Nut 6

Start of Block: VET/NUT 7

NUTRITION

Q31 Do you recommend modifying feed strategies during extreme heat events?

- ◯ Yes (1)
- ◯ No (2)

Display This Question:

If Do you recommend modifying feed strategies during extreme heat events? = Yes

Q31.2 If you recommend modifying feeding strategies during extreme heat events, please answer yes or no:

- ▢ Do you alter feed composition? (1) ________________
- ▢ Do you change feed delivery times? (2) ________________
- ▢ Do you utilize different feed additives during heat stress events? (3) ________________

Q32 Do you recommend modifying feed strategies after extreme heat events to help cattle recover?

◯ Yes (1)

◯ No (2)

Display This Question:

If Do you recommend modifying feed strategies after extreme heat events to help cattle recover? = Yes

Q32.2 If you recommend modifying feeding strategies after extreme heat events please answer yes or no:

☐ Do you alter feed composition? (1)

☐ Do you change feed delivery times? (2)

☐ Do you utilize different feed additives during heat stress events? (3)

End of Block: VET/NUT 7

Start of Block: VET/NUT 8

PERCEPTIONS

Q33 Please rate the following on a scale from "not effective" (0) to "extremely effective" (10) as it relates to minimizing the effects of heat stress on cattle. Please check not applicable if you do not know.

Not Applicable

0	1	2	3	4	5	6	7	8	9	10

Changing water availability ()	
Changing feeding strategies ()	
Providing shade ()	
Changing processing and shipping hours ()	
Using a sprinkler system ()	

Q34 Please rate the following on a scale from "strongly disagree" (0) to "strongly agree" (10). Please check not applicable if you do not know.

Not Applicable

0 1 2 3 4 5 6 7 8 9 10

Heat stress negatively impacts cattle performance. ()	
Heat stress negatively impacts cattle health. ()	
Heat stress negatively impacts cattle welfare. ()	
Heat stress negatively impacts carcass quality. ()	
The triggers I use to begin implementing heat stress mitigation work. ()	
The heat stress monitoring tools I use are helpful. ()	
Involving a veterinarian in heat stress management decisions is helpful. ()	
Involving a nutritionist in heat stress management decisions is helpful. ()	

Q35 Are there any additional comments you would like to provide

____

Q36 What are some obstacles in implementing heat stress mitigation strategies? Select all that apply.

- ▢ Cost (1)
- ▢ Expertise (2)
- ▢ Facility limitations (3)
- ▢ Labor (4)
- ▢ Natural resources (e.g., water) (5)
- ▢ Time (6)
- ▢ Training (7)
- ▢ Scheduling (8)
- ▢ Lack of data to demonstrate effectiveness (9)
- ▢ Other, please explain. (10) ____

Q37 What resources would be helpful to you in regards to managing heat stress? Please explain.

____

End of Block: VET/NUT 8

Start of Block: Block 9

EXTREME HEAT EVENTS

Q38 Have you experienced cattle death loss from extreme heat events?

- ○ Yes (1)
- ○ No (2)

Display This Question:

If Have you experienced cattle death loss from extreme heat events? = Yes

Q38.2 How frequently do you experience cattle death loss from extreme heat events?

- ○ Multiple times per year (1)
- ○ Once per year (2)
- ○ Every few years (3)

Display This Question:

If Have you experienced cattle death loss from extreme heat events? = Yes

Q38.3 During which months of the year have you experienced extreme heat events?

- ▢ April (4)
- ▢ May (5)
- ▢ June (6)
- ▢ July (7)
- ▢ August (8)
- ▢ September (9)
- ▢ Other (12) ____

Display This Question:

If Have you experienced cattle death loss from extreme heat events? = Yes

Q38.4 Would you consider your loss as:

- ○ Minor (5)
- ○ Moderate (6)
- ○ Severe (7)

Display This Question:

If Would you consider your loss as: = Minor

Or Would you consider your loss as: = Moderate

Or Would you consider your loss as: = Severe

Q38.4.2 Please explain your selection (minor, moderate or sever) for your loss during extreme heat events (e.g, amount of cattle lost, impact on employees)

____

Q39 What do you consider the class(es) of animals with the highest risk for death loss?

____

Q40 Please share what you would do differently in the face of another extreme heat event.

____

End of Block: Block 9

Start of Block: Block 10

DEMOGRAPHICS

Q41 What gender do you identify with?

- ○ Male (1)
- ○ Female (2)
- ○ Other (3)
- ○ Prefer not to answer (4)

Q42 How would you best describe yourself? Select all that apply.

- ☐ American Indian or Alaska Native (1)
- ☐ Asian (2)
- ☐ Black or African American (3)
- ☐ Native Hawaiian or Other Pacific Islander (4)
- ☐ White (5)
- ☐ Other, please specify: (6) ____
- ☐ Prefer not to answer (7)

Q43 Do you identify as Hispanic, Latino/a/x or Spanish origin?

- ○ Yes (1)
- ○ No (2)
- ○ Other, please specify: (3) ____
- ○ Prefer not to answer (4)

Q44 What is your age (in years)?

____

Q45 Approximately how many years have you been working in the cattle industry?

- ○ Less than 1 year (1)
- ○ 1 to 5 years (2)
- ○ 5 to 10 years (3)
- ○ >10 years (4)

End of Block: Block 10
